# Lessons learned during down referral of antiretroviral treatment in Tete, Mozambique

**DOI:** 10.1186/1758-2652-12-6

**Published:** 2009-05-06

**Authors:** Tom Decroo, Isabella Panunzi, Carla das Dores, Fernando Maldonado, Marc Biot, Nathan Ford, Kathryn Chu

**Affiliations:** 1 Médecins Sans Frontières, Tete, Mozambique; 2 Provincial Health Department, Tete, Mozambique; 3 Médecins Sans Frontières, Maputo, Mozambique; 4 South African Medical Unit, Médecins Sans Frontières, Johannesburg, South Africa

## Abstract

As sub-Saharan African countries continue to scale up antiretroviral treatment, there has been an increasing emphasis on moving provision of services from hospital level to the primary health care clinic level. Delivery of antiretroviral treatment at the clinic level increases the number of entry points to care, while the greater proximity of services encourages retention in care.

In Tete City, Mozambique, patients on antiretrovirals were rapidly down referred from a provincial hospital to four urban clinics in large numbers without careful planning, resulting in a number of patients being lost to follow-up.

We outline some key lessons learned to support down referral, including the need to improve process management, clinic infrastructure, monitoring systems, and patient preparation. Down referral can be avoided by initiating patients' antiretroviral treatment at clinic level from the outset.

## Introduction

As sub-Saharan African countries continue to scale up antiretroviral treatment (ART), there has been an increasing emphasis on moving provision of services from hospitals to primary heath care (PHC) clinics. Decentralization is an important strategy for improving access to ART, particularly in rural areas. Decentralization is defined as the process of moving delivery of ART from hospitals to clinics, thereby improving access (proximity) to care, and encouraging greater retention in care (less defaulting) [1-3].

A recent review of loss to follow-up in ART programmes in resource-limited settings concluded that retention rates are better in services that have smaller numbers of patients and that population coverage should be supported by smaller decentralized facilities rather than by a few large programmes [4].

Despite the logic and evidence that supports the decentralization of HIV/AIDS care to the PHC level, in many settings, HIV care is provided only at hospital level. As hospital services become saturated, there will be a need to "down refer" patients to lower levels of the health system.

However, this process of down referral must be carefully planned and executed to avoid overwhelming primary care services and to maximize patient retention. Reports from TB programmes have shown that almost a third of patients are lost on referral between the hospital and clinic [5]. This article describes the lessons learned from a large-scale down referral of ART services in Tete, Mozambique.

## Context

Tete City (population c.170,000) is the capital of the Tete Province in central Mozambique, and has an adult HIV prevalence of 19% (± 5%) [6,7]. In 2003, the Provincial Health Department, with the support of Médecins Sans Frontières (MSF), began an ART programme at Tete Provincial Hospital in Tete City. At that time, this was the only health facility that provided ART in the province.

By the end of 2006, 2,350 patients had been initiated on ART at Tete Provincial Hospital, of which 1,637 (70%) remained in care, resulting in an overwhelming patient load at hospital level. Only 218 patients had been initiated on ART in the four urban PHC clinics by the end of that year.

In May 2007, the hospital directorate issued a mandate to down refer around 1,000 patients (61%) followed at the provincial hospital. This was in preparation for major reconstruction work at the hospital, which resulted in a six-fold reduction in consultation rooms for HIV.

**Figure 1:**
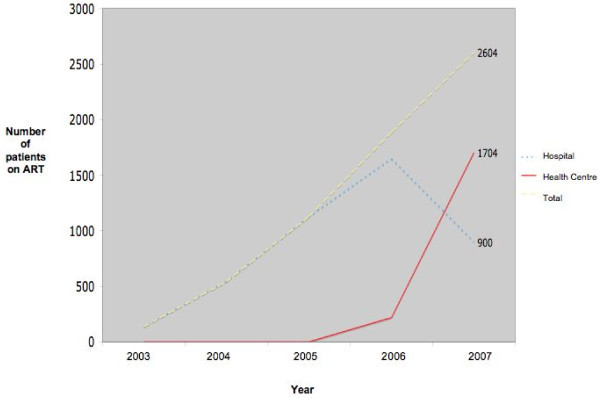
**Number of patients enrolled on ART care in Tete hospital and PHC clinics**.

Due to the inadequate capacity of the clinics to manage so many new patients at that time, only stable patients (those on ART for at least six months and with no clinical complications) were considered for down referral. Within nine months, the number of patients managed at the clinics increased nearly eight-fold, from 218 to 1,704 patients (Figure 1).

While down referral is a necessary step to integrate HIV/ART services into the PHC system, this process in Tete was particularly challenging. This was due to its rapid implementation, which resulted in more than 100 patients being lost to care (representing a loss to follow up rate of 30%). Capacity and human resources at the PHC level were rapidly overwhelmed until structural and human resource solutions were proposed and implemented.

## Challenges

### Management

The down referral process began before the completion of planning with all involved stakeholders because of pressure to implement the decision to down refer. Staff at the PHC clinics, although trained in ART care, did not fully appreciate the extent of the services that would need to be provided, and were not experienced enough to manage the large influx of patients on ART.

Although the criteria for down referral were well defined, some non-eligible patients were also down referred in the drive to move patients out of the hospital. Finally, too many patients were referred at once, instead of a phased approach being implemented.

### Primary health care clinic infrastructure

As a result of the influx of patients, the overall number of consultations (HIV and non-HIV) at each clinic more than doubled. Initially, each PHC clinic had only one ART consultation room. Consequently, other rooms, such as sterilization areas or changing rooms, were used for consultations.

Waiting time was often several hours, and waiting areas became overcrowded. The increased patient load put a severe strain on other clinic services: laboratories could not keep up with the increase in blood collections; and pharmacies became congested with long waiting lines as insufficient staff members were trained to dispense antiretrovirals (ARVs).

Drug supply management also became a problem as PHC clinics were not aware of the extent of referral numbers to expect and could not forecast consumption. When medications ran out, patients returned to the hospital to fill their prescriptions. Some patients initially asked to be transferred back to the hospital due to frustration with the chaotic process.

### Patient monitoring and data management

The transfer of patients between the hospital and the clinics was not well monitored. With many providers down referring, there was no master list of all patients to be transferred. Referral letters were supposed to accompany patients, but these were sometimes lost. The electronic database maintained at the hospital was not updated when patients were down referred.

No systematic active tracing of these "loss to follow-up" patients was conducted until the following year. Lists of "loss to follow-up" patients were sent to the clinics in an attempt to trace and update the database, but the clinics had meanwhile issued new patient identification numbers, so records did not match. Therefore, the true outcomes for those patients lost during referral remain unknown. As these were stable patients with no clinical complications, mortality is unlikely to have contributed to this rapid attrition over a short time period [8].

### Patient education

In the beginning, some patients were reluctant to be down referred as they did not fully understand the advantages (easier access to services) and disadvantages (less confidentially due to closeness to their community) of follow-up at the PHC clinics. Consequently, they feared a decrease in the quality of care; several patients refused to be down referred or they decided, without informing the medical team, to self-transfer from one clinic to another.

## Proposed solutions

The hospital and clinic staff, along with the provincial health department, identified the problems described here, and jointly proposed a number of actions (Table 1). A joint MSF and provincial health team, dedicated to assisting the clinics with the down referral process, was assembled.

**Table 1:** Essential steps in down referral of HIV/ART services from hospital to primary health care clinic level

Planning
Joint hospital, primary level care staff and patient representatives to discuss feasibility of down referral
Down referral criteria established
Phased implementation according to capacity
Establish dedicated team who will oversee down referral process



**Primary health clinic human resources and infrastructure**

Well trained and adequate number of clinicians
Continued coaching and training during down referral
Task shifting
Receptionists and data managers to accurately register and track patients
Adequate clinic space (i.e. consultation rooms and pharmacy)
Ensure adequate supply of antiretroviral medications
Peer counsellors trained at hospital and PHC level on how to negotiate process of down referral with service users and service providers



**Patient flow and education**

Improve efficiency of patient care by establishing fast track and designated phlebotomy dates
Implement appropriate and simplified data collection tools
Standardize identification numbers between tertiary and primary care centres so tracing would be easier
Establish regular contact between tertiary and primary levels to ensure all transferred patients are enrolling at PHC level
Conduct lost to care tracing of patients who are down referred but are subsequently "lost"
Train counsellors at the tertiary and PHC level on how to educate patients on the process of the down referral

This team oversaw a number of actions, including the establishment of monthly quotas of patients to be down referred to prevent overwhelming the clinics. The actions allowed for: better stock forecasting; reorganization of clinic laboratories so that routine blood collection was done on specific days; training in stock management for the PHC pharmacists; and establishment of a buffer stock of ARVs and medications to treat opportunistic infections in case clinic stocks became depleted.

Human resources were also restructured: two nurses were moved from the hospital to the clinics, and receptionists were hired to register patients at the clinics and collect demographic data. The latter is an example of "task shifting" of work previously done by the clinicians or counsellors [9].

To improve patient flow, a fast track system was created for stable patients so that they only needed to come to the clinic every three months. A simplified data collection system was implemented for monitoring and evaluation, including the use of check lists of patients to be down referred and of those who actually registered at the PHC clinics. Finally, counsellors at the hospital were trained to explain the reasons for the down referral, while counsellors in the PHCs were trained to receive the referred patients.

## Conclusion

In other settings in southern Africa, decentralization has proven to be a successful strategy for supporting scale-up of antiretroviral therapy [1,3,10-12]. In particular, providing ART at the PHC clinic level increases the number of entry points to care, while the greater proximity of services encourages retention in care [1,4].

In Tete City, the majority of clients referred to clinic services continued their follow-up at clinic level. Down referral was, in the end, broadly accepted as these services were more accessible.

However, a number of issues should be considered to ensure that appropriate support is given to PHC clinics. The mass transfer of patients enrolled in care at the hospital level can quickly overwhelm minimally staffed clinics if appropriate steps are not taken. In addition to the extra workload, clinic staff may feel uncomfortable with their new level of responsibility, particularly if training and supervision mechanisms are not in place. The Tete experience serves to highlight a number of simple steps that can be taken to ensure a smooth transition from hospital-based to clinic-based care.

The short-term chaos has been outweighed by the broader benefits of establishing a decentralized programme. As of December 2008, more than 2,700 patients on ART were being followed in the four PHC clinics, compared to around 800 in the hospital.

Most problems during down referral were successfully resolved through the creation of a team that worked across different areas of the health service to address a range of challenges, from drug supply to human resources. At the same time, a number of changes were made to reinforce the capacity and efficiency of the primary health care clinics.

Down referral requires careful planning, implementation over a realistic timeframe, and attention to monitoring at all levels. Perhaps the most obvious lesson is the need to take time to explain to the patients the reasons behind the decisions taken for the down referral, and explain that they would benefit from more proximal services without any compromise in care. Criteria for referral should ideally be determined in consultation with all stakeholders, including service users.

Finally, given the growing evidence that most ART cases can be initiated at clinic level, the problems associated with down referral could have been avoided by initiating newly enrolled patients directly at PHC clinic level from the outset.

## Competing interests

The authors declare that they have no competing interests.

## Authors' contributions

MB, TD, and KC provided the initial conception and design. FM and IP analyzed the data. All authors contributed to the interpretation and discussion of the data. KC and IP drafted the article. MB, TD, CD and NF provided critical revision of the article for important intellectual content. The final version of the manuscript was seen and approved by all authors.
